# Non-invasive assessment of acute heart failure by Stevenson classification: Does echocardiographic examination recognize different phenotypes?

**DOI:** 10.3389/fcvm.2022.911578

**Published:** 2022-09-27

**Authors:** Alberto Palazzuoli, Gaetano Ruocco, Serafina Valente, Andrea Stefanini, Erberto Carluccio, Giuseppe Ambrosio

**Affiliations:** ^1^Cardiovascular Diseases Unit, Cardio Thoracic Department, Le Scotte Hospital, University of Siena, Siena, Italy; ^2^Cardiology Unit, Riuniti of Valdichiana Hospitals, USL-SUD-EST Toscana, Montepulciano, Italy; ^3^Cardiology Unit, Cardio Thoracic Department, Le Scotte Hospital, University of Siena, Siena, Italy; ^4^Division of Cardiology and Center for Clinical and Translational Research – CERICLET, University of Perugia, Perugia, Italy

**Keywords:** Stevenson classification, acute heart failure (AHF), congestion, perfusion, echocardiography

## Abstract

**Background:**

Acute heart failure (AHF) presentation is universally classified in relation to the presence or absence of congestion and the peripheral perfusion condition according to the Stevenson diagram. We sought to evaluate a relationship existing between clinical assessment and echocardiographic evaluation in patients with AHF.

**Materials and methods:**

This is a retrospective blinded multicenter analysis assessing both clinical and echocardiographic analyses during the early hospital admission for AHF. Patients were categorized into four groups according to the Stevenson presentation: group A (warm and dry), group B (cold and dry), group C (warm and wet), and group D (cold and wet). Echocardiographic evaluation was executed within 12 h from the first clinical evaluation. The following parameters were measured: left ventricular (LV) volumes, LV ejection fraction (LVEF); pattern Doppler by E/e1 ratio, pulmonary artery systolic pressure (PASP), tricuspid annular plane systolic excursion (TAPSE), and inferior cave vein diameter (ICV).

**Results:**

We studied 208 patients, 10 in group A, 16 in group B, 153 in group C, and 29 in group D. Median age of our sample was 81 [69–86] years and the patients enrolled were mainly men (66.8%). Patients in groups C and A showed significant higher levels of systolic arterial pressures with respect to groups B and D (respectively, 130 [115–145] mmHg vs. 122 [119–130] mmHg vs. 92 [90–100] mmHg vs. 95 [90–100] mmHg, *p* < 0.001). Patients in groups A and C (warm) demonstrated significant higher values of LVEF with respect to patients in groups B and D (43 [34–49] vs. 42 [30–49] vs. 27 [15–31] vs. 30 [22–42]%, *p* < 0.001). Whereas group B experienced significant lower TAPSE values compared with other group (14 [12–17] mm vs. A: 17 [16–21] mm vs. C: 18 [14–20] mm vs. D: 16 [12–17] mm; *p* = 0.02). Finally, echocardiographic congestion score including PASP ≥ 40 mmHg, ICV ≥ 21, mm and E/e’ > 14 did not differ among groups. Follow-up analysis showed an increased mortality rate in D group (HR 8.2 *p* < 0.04).

**Conclusion:**

The early Stevenson classification remains a simple and universally recognized approach for the detection of congestion and perfusion status. The combined clinical and echocardiographic assessment may be useful to better define the patients’ profile.

## Introduction

Acute heart failure (AHF) comprehends a wide clinical presentation due to underlying conditions and disease substrate, as well as to congestion and perfusion status (1). These features are directly related to systemic fluid retention and cardiac pump capacities. All conditions causing structural or functional cardiac abnormalities resulting in reduced cardiac output and/or elevated left ventricular (LV) filling pressures (LVFP) are potential triggers for both fluid accumulation and or reduced peripheral perfusion (2, 3). Systemic vasoconstrictions associated with low perfusion are often a consequence of reduced stroke volume or elevated filling pressure. Arterial-venous oxygen content difference is another determinant of the oxygen delivery to the tissue and it directly modulates vascular status. Low cardiac output and cardiac index below ≤1.8 l/min/m^2^ are usually associated with extremely poor prognosis, demanding prompt and effective management to raise systemic blood pressure and to restore adequate perfusion (4, 5). Similarly, high congestion burden and degree are associated with adverse outcome (6, 7). However, poor agreement has been demonstrated between congestion status and hemodynamic evaluation (8). According to this paradigm, ESC HF 2016 guidelines highlight the importance to identify a precise clinical and hemodynamic status to customize the appropriate treatment and improve outcome (9). In 1976, first, Forrester et al. demonstrated that among patients with acute myocardial infarction, invasive catheterization identified four hemodynamic profiles based on the presence or absence of congestion (PCWP > or ≤ 18 mmHg) and adequacy of perfusion (CI > 2.2 l/min/m^2^) (10). Subsequently, Stevenson et al. confirmed four HF subtypes according to clinical characteristics defined by the absence or presence of symptoms and signs of congestion and by the evidence of adequate or inadequate perfusion signs (7, 9). This clinical algorithm combines the clinical examination with the assessment of some hemodynamic parameters, such as blood pressure and heart rate. Additionally, it is a clinical algorithm including the signs of congestion and perfusion capable of identifying patients with higher risk for adverse events. Unfortunately, there is a lack of concordance between invasive and clinical measurement, and few studies reporting contemporarily the evaluation of clinical and hemodynamic status during early hospital admission have been published before (11, 12).

Despite this universally recognized schematic approach, the diagnosis of AHF typology based on clinical features alone may imply considerable diagnostic uncertainty. Additional diagnostic information is often necessary to support the clinicians in characterizing the status and type of patients with AHF (13). Thus, a bedside echocardiographic examination may help in HF definition and Stevenson re-classification (14) and echocardiographic examination measuring certain parameters became an additional tool for a more precise clinical evaluation (15). Following these observations, we aim to study 1– the relation existing between clinical sign of congestion or perfusion in accordance with Stevenson classification and echocardiographic parameters 2– the capacity of systematic echocardiographic analysis to reclassify and change the initial clinical presentation.

## Materials and methods

This is a retrospective multicenter case–control analysis of three Italian hospitals Cardiovascular Disease Unit Internal Medicine Department, Cardiology Unit Cardio-Thoracic Department (Siena) and Cardiology Unit University of Perugia including consecutive patients with AHF consecutively enrolled from December 2018 to November 2021. All patients were defined according to the last ESC criteria encountering at least two indicators among the clinical functional imaging and historical variables (9, 11). All patients were in advanced III or IV NYHA class, requiring intravenous dose of furosemide. The Stevenson classification scheme is based on bedside evaluation and categorization by clinical signs of congestion (“wet” vs. “dry” if present vs. absent) and hypoperfusion (“cold” vs. “warm” if present vs. absent): It recognizes four distinct profiles: “dry-warm” – free of either congestion or hypoperfusion “wet-warm” – patients demonstrating congestion and adequate peripheral perfusion; “dry-cold” – free of congestion but with hypoperfusion; “wet-cold” – with congestion and hypoperfusion.

Patients were enrolled within 12 h of hospital admission because of a diagnosis of new onset or decompensated ADHF, based on the signs and symptoms of ADHF and elevated levels of natriuretic peptides (B-type natriuretic peptide [BNP] > 100 pg/ml or aminoterminal pro B-type natriuretic peptide [NTpro BNP] > 300 pg/ml). Patients were defined as having HFrEF if left ventricular ejection fraction (LVEF) was <50%, and HFpEF if LVEF was ≥50%. We excluded from the study patients performing echo after the first 12 h from admission, patients previously submitted to inotropic or infusive administration, patients with recent heart valve replacement, recent coronary artery bypass graft (<3 months), history of pulmonary embolism, idiopathic pulmonary arterial hypertension, neoplastic, hematologic and immune diseases with systemic involvement, and patients with a history of pneumothorax and/or lobectomy.

### Patients evaluation

Patients were evaluated at admission by two physicians assessing the degree of clinical congestion giving 1 point for each of the following signs: orthopnea, pulmonary rales, third heart sound, jugular venous distension, peripheral edema, and hepatomegaly (6 points) (13). *Hemodynamic parameters (heart rate and blood pressure) were also assessed*. Adequate or inadequate perfusion was evaluated by the occurrence of hypotension (systolic blood pressure value <90 mmHg) tachycardia associated with pulsus alternans, cold extremities, decreased urine output, dizziness, and narrow pulse pressure (6 points). We categorized our patients by the contemporary presence of at least 2 signs of congestion and hypoperfusion, respectively (11, 16).

### Echocardiography

Echocardiography was performed at hospital admission *before patients start the treatment* by physicians who were unaware of the clinical assessment. All the examinations were performed by cardiologists according to the instructions provided by the American Society of Echocardiography (17). The main measurements were recorded and independently reviewed by two distinct physicians; systolic and diastolic left ventricular volumes and ejection fraction were determined using apical two- and four-chamber views by Simpson biplane formula. The following parameters were obtained at pulsed Doppler transmitral flow velocity: early diastolic velocity (E wave), late diastolic velocity (A wave) and their ratio (E/A), and deceleration time (DT) of E wave. Early diastolic myocardial velocity (e’) was obtained at tissue Doppler imaging (TDI) at septal level and the E/e’ ratio was calculated. TAPSE was obtained by placing the M-mode cursor laterally to the tricuspid annulus. PASP was estimated by continuous Doppler at tricuspid valve level, as the sum of 4* peak velocity of tricuspid regurgitation and the estimate of right atrial pressure based on inferior vena cava diameter and collapsibility. The right ventricular end-diastolic diameter (RVEDD) was measured by apical for chamber view at basal level below tricuspid annulus.

Severe diastolic dysfunction was defined as follows: a DT of the E-wave <140 ms and E/e’ ratio >14 and it was accounted as sign of increased LV filling pressure (18). Patients were categorized as having high PASP if the PASP estimate was ≥ 40 mmHg. The cutoff for RV dysfunction was set at a TAPSE value of ≤16 mm (19). We focused analysis on 6 main parameters to compare echocardiographic finding with Stevenson presentation: left ventricular (LV) volumes, LV ejection fraction (LVEF); E/e’ ratio, pulmonary artery systolic pressure (PASP), tricuspid annular plane systolic excursion (TAPSE), and inferior cave vein diameter (ICV).

### Follow-up

The primary outcome was the composite of all-cause mortality and/or HF or cardiovascular events re-hospitalization. All patients were followed after discharge for 30-day adverse events occurrence. Adverse events considered in the follow-up were deaths for heart failure or cardiovascular causes, heart failure hospitalizations, acute coronary syndromes, ventricular or supraventricular arrhythmias, or heart failure associated with worsening renal function.

### Statistical analysis

Categorical data were presented as numbers and percentages and were analyzed with the chi-square test; continuous variables were shown as median and interquartile range [IQR] because of non-normally distributed. Patients with AHF were grouped by perfusion (warm and cold) and congestion (wet and dry) in four groups and the differences among the four groups were analyzed with the Kruskal–Wallis test. The Mann–Whitney test was employed for continuous variables analysis if two groups were compared. Kaplan–Meier curves with the log-rank statistics were used to illustrate event rates at the time point of interest for the four groups. We considered statistically significant results associated with a *p* ≤ 0.05. We used the SPSS software (version 20.0) for all analyses.

## Results

**FIGURE 1:**
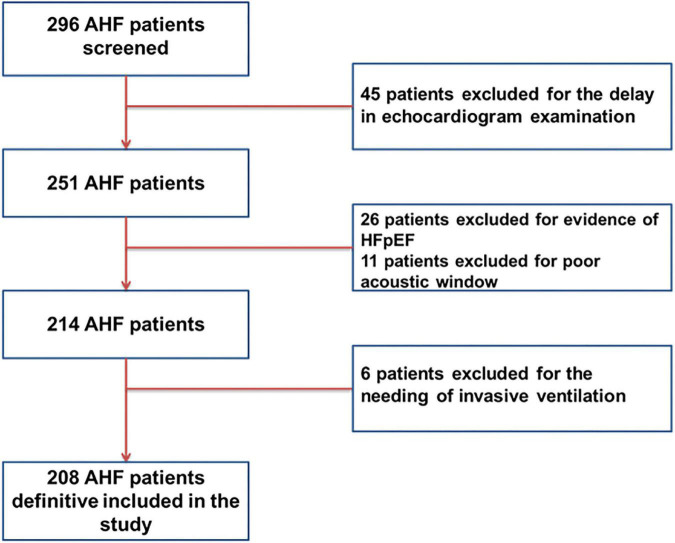
Flow chart of the patients included in the study divided according to Stevenson profile.

This study included 208 patients admitted in hospital for AHF *divided by clinical and hemodynamic profiles* (Figure 1). Our population was divided into four groups according to Stevenson classification: 10 (5%) patients defined as warm and dry (group A), 16 (7.7%) patients defined as cold and dry (group B), 153 (73.3%) patients defined as wet and warm (group C), and 29 (14%) patients defined as cold and wet (group D). Median age of our sample was 81 [69–86] years and the patients enrolled were mainly men (66.8%).

In our population, median NTproBNP and estimated glomerular filtration rate (eGFR) were, respectively, 7,777 [4,200–14,014] pg/ml and 45 [31.6–54] ml/min/m^2^. Median of systolic arterial pressure and diastolic arterial pressure was 120 [105–140] and 70 [60–80] mmHg. Echocardiographic analysis revealed that in all patients, the median of LVEF, TAPSE, PASP, and ICV was, respectively, 38 [27–49]%, 17 [14–20] mm, 45 [35–53] mmHg, and 20 [16–24] mm.

The analysis of clinical, demographic, and echocardiographic characteristics in the four groups demonstrated that there were not significant differences among groups in terms of NTproBNP, eGFR, diabetes, dyslipidemia, chronic kidney disease (CKD), LV diameters and volumes, ICV, and E/e’. However, patients in group C (warm and wet) were older with respect to patients in groups A, B and D (82 [70–87] vs. 78 [63–81] vs. 73 [58–81]vs. 79 [66–88] years; *p* = 0.058). Moreover, patients in group C and A showed significant higher levels of both systolic and diastolic arterial pressures with respect to groups B and D (respectively, 130 [115–145] vs. 122 [119–130] vs. 92 [90–100] vs. 95 [90–100] mmHg, *p* < 0.001; 75 [70–84] vs. 70 [64–81] vs. 60 [55–64] vs. 60 [53–60] mmHg, *p* < 0.001).

Pulmonary artery systolic pressure values were higher in patients of group C compared to other groups (groups A, B, and D) (45 [35–55] vs. 32 [29–46] vs. 42 [36–49] vs. 45 [36–54] mmHg, *p* = 0.097), whereas group B experienced significant lower TAPSE values compared with other groups (14 [12–17] vs. A: 17 [16–21] vs. C: 18 [14–20] vs. D: 16 [12–17] mm; *p* = 0.02).

**FIGURE 2:**
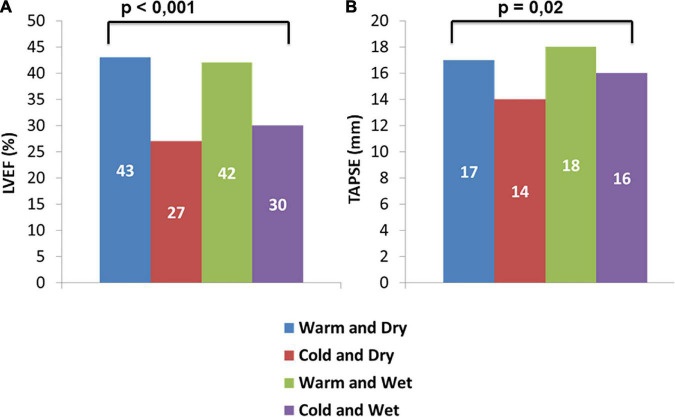
Difference in median of LVEF **(A)** and TAPSE **(B)** among AHF patients’ groups.

Patients in groups A and C (warm) demonstrated significantly higher values of LVEF with respect to patients in groups B and D (43 [34–49] vs. 42 [30–49] vs. 27 [15–31] vs. 30 [22–42]%, *p* < 0.001; Table 1 and Figure 2).

**TABLE 1:** Demographic, clinical, and echocardiographic differences among warm/cold and wet/dry groups.

1	AHF patients presentation phenotypes (no. of patients)					
		
Variables	All patients (208)	A (10)	B (16)	C (153)	D (29)	*P*-value
Age (years)	81 [69–86]	78 [63–81]	73 [57–84]	82 [70–87]	79 [66–88]	0.049
Gender Male (%)	66.8	80	87.5	64.7	62.1	0.215
*CV risk factors (%)* Hypertension	58.7	80	25	69.3	13.8	<0.001
Diabetes	38.5	50	31.2	37.3	44.8	0.681
Dyslipidemia	54.8	60	43.8	55.6	55.2	0.817
Smoking CKD *NTproBNP (pg/mL)* *eGFR (ml/min/m^2^)* *Echocardiography*	45.2 43.8 7,777[4,200–14,014] 45 [31.6–54]	50 50 5,285 [2,246–25,895] 45 [40.7–50]	43.8 37.5 7,530 [3,420–26,837] 47.5 [35.2–58]	43.8 47.1 7,800 [4,200–13,392] 46 [31–54]	51.7 27.6 8,430 [5,096–21,690] 40 [28.4–56.5]	0.867 0.243 0.569 0.816
LVEF (%) LVEDD (mm) LVESD (mm) LVEDV (ml) LVESV (ml) TAPSE (mm) ICV (mm) PASP (mmHg) E/e’	38 [27–49] 58 [49–64] 44 [34–50] 140 [100–180] 95 [55–120] 17 [14–20] 20 [16–24] 45 [35–53] 15 [12–18]	43 [34–49] 55 [47–58] 39 [33–43] 166 [115–142] 86 [55–84] 17 [16–21] 17 [16–21] 32 [29–46] 16 [13–18]	27 [15–31] 62 [55–71] 49 [36–56] 130 [104–212] 97 [70–149] 14 [12–17] 21 [18–26] 42 [36–49] 14 [12–18]	42 [30–49] 58 [49–64] 43 [34–50] 140 [100–180] 90 [51–117] 18 [14–20] 20 [17–24] 45 [35–55] 15 [12–18]	30 [22–42] 57 [49–70] 44 [33–51] 137 [109–164] 100 [65–122] 16 [12–17] 22 [15–23] 45 [36–54] 15 [11–18]	<0.001 0.349 0.366 0.769 0.684 0.020 0.307 0.097 0.623
Systolic arterial pressure Diastolic arterial pressure	120 [105–140] 70 [60–80]	122 [119–130] 70 [64–81]	92 [90–100] 60 [55–64]	130 [115–145] 75 [70–84]	95 [90–100] 60 [53-60]	<0.001 <0.001

A, warm and dry; B, cold and dry; C, warm and wet; D, cold and wet. CV, Cardiovascular; CKD, chronic kidney disease; eGFR, estimated glomerular filtration rate; ICV, inferior cave vein; LVEDD, left ventricular end-diastolic diameter; LVEDV, left ventricular end-diastolic volume; LVEF, left ventricular ejection fraction; LVESD, left ventricular end-systolic diameter; LVESV, left ventricular end-systolic volume; NTproBNP, aminoterminal pro B-type natriuretic peptide; PASP, pulmonary artery systolic pressure; TAPSE, tricuspid annular plane systolic excursion. The bold were employed for group definition.

Dividing our sample into two groups according to perfusion status, we observed that patients in “*warm*” group had significantly higher values of LVEF (42 [30–49] vs. 29 [20–39]%; *p* < 0.001) and TAPSE (18 [14–20] vs. 16 [1–217] mm; *p* = 0.002) compared to “*cold*” group (Table 2A). Conversely, dividing our sample into two groups in relation to congestion status (wet and dry), we foundsignificantly higher values of both PASP and LVEF in “*wet*” group (PASP: 45 [35–55] vs. 40 [30–46] mmHg; *p* = 0.029. LVEF: 40 [28–49] vs. 30 [20–46]%; *p* = 0.021) with respect to “*dry*” groups (Table 2B).

**TABLE 2:** Echocardiographic differences among warm and cold patients (A) and wet and dry patients (B).

AHF patients perfusion status (A)			
Variables	Warm (163)	Cold (45)	*P*-value
LVEF (%) LVEDD (mm) LVESD (mm) LVEDV (ml) LVESV (ml) TAPSE (mm) ICV (mm) PASP (mmHg) E/e’	42 [30–49] 58 [49–64] 42 [34–50] 140 [100–180] 90 [52–114] 18 [14–20] 20 [16–24] 45 [35–55] 15 [12–18]	29 [20–39] 60 [49–70] 45 [35–55] 135 [106–181] 99 [70–127] 16 [12–17] 21 [16–24] 45 [36–50] 14 [12–18]	<0.001 0.218 0.323 0.640 0.224 0.002 0.799 0.942 0.190

**AHF patients congestion status (B)**			

**Variables**	**Wet (182)**	**Dry (26)**	***P*-value**

LVEF (%) LVEDD (mm) LVESD (mm) LVEDV (ml) LVESV (ml) TAPSE (mm) ICV (mm) PASP (mmHg) E/e’	40 [28–49] 58 [49–65] 44 [34–50] 140 [100–180] 95 [54–120] 17 [14–20] 20 [16–24] 45 [35–55] 15 [12–18]	30 [20–46] 58 [50–63] 43 [36–51] 140 [110–202] 97 [71–132] 16 [14–19] 19 [17–25] 40 [30–46] 15 [12–18]	0.021 0.856 0.853 0.502 0.633 0.312 0.933 0.029 0.769

ICV, inferior cave vein; LVEDD, left ventricular end-diastolic diameter; LVEDV, left ventricular end-diastolic volume; LVEF, left ventricular ejection fraction; LVESD, left ventricular end-systolic diameter; LVESV, left ventricular end-systolic volume; PASP, pulmonary artery systolic pressure; TAPSE, tricuspid annular plane systolic excursion. The bold were employed for group definition.

**FIGURE 3:**
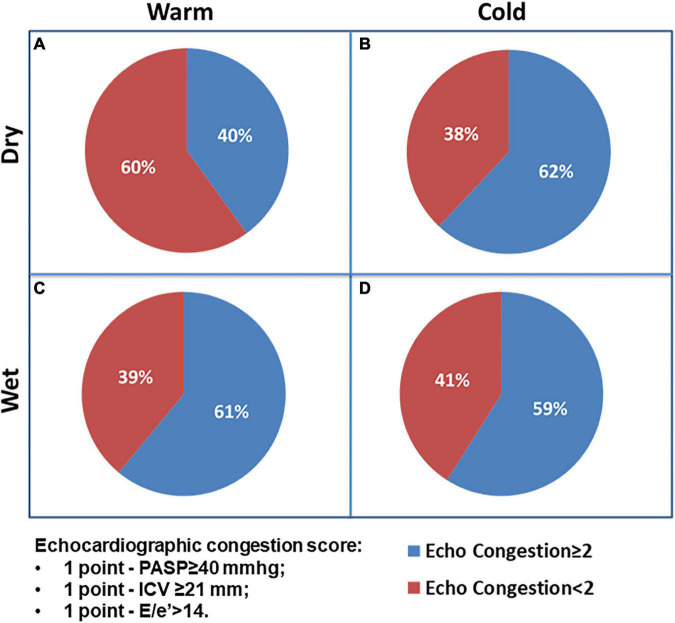
The Chi-square test for prevalence of echocardiographic congestion score ≥ 2 among AHF patients’ groups.

The analysis of echocardiographic congestion score includes 1 point for each of the following variables: PASP ≥ 40 mmHg, ICV ≥ 21 mm, and E/e’ > 14, and the prevalence of patients with echocardiographic score ≥ 2 did not significantly differ among groups (A: 40% vs. B: 62% vs. C: 61% vs. D: 59%; *p* = 0.601). Current findings imply that a significant percentage clinically judged as not congested experienced echo signs of congestion (Figure 3).

**FIGURE 4:**
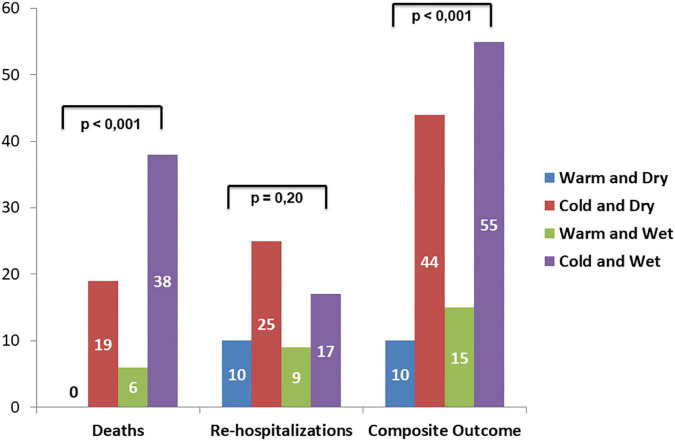
Adverse events rate among AHF patients’ groups according to Stevenson presentation: left bars show death, middle bars refer to re-hospitalization, and right bars refer to the composite outcome.

**FIGURE 5:**
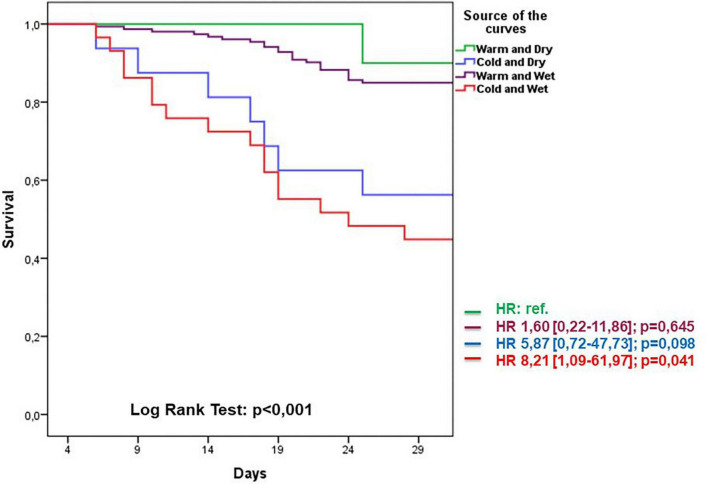
Kaplan–Meier survival curves related to AHF presentation during 30-day follow-up period groups. Warm and dry (green) cold and dry (blue) warm and wet (violet) cold and wet (red).

Follow-up data demonstrated a total of 47 adverse events at 30 days of which 23 deaths and 24 re-hospitalizations. Death rate was significantly increased in wet and cold groups with respect to other groups. The cold groups (B and D) showed a worse outcome with respect to the warms (HR 5.8 and 8.2, respectively). Re-hospitalizations rate did not demonstrate significant differences among groups (Figure 4). Kaplan–Meier survival curves showed that wet and cold AHF presentation was significantly related to poor prognosis (Figure 5).

## Discussion

### Main findings

This is the first study directly comparing clinical presentation classified by Stevenson formulation recognizing four principal subtypes, with a simple echocardiographic score. We report that congestive status is usually recognized by the increased PASP and ICV values; however, a significant percentage defined as “dry” patients have some signs of echocardiographic congestion in terms of E/e’ ratio increase and PASP elevation, despite clinical examination does not reveal sign of congestion. Interestingly, “cold” status reflecting hypoperfusion profile reveals reduced TAPSE and lower EF compared with other groups implying poor RV and LV systolic function, respectively. The reference group with no congestion and no hypoperfusion (group A dry and warm) demonstrated lower echocardiographic congestion signs in terms of PASP VCI and diastolic filling pattern. The most important findings of our analysis are the underestimation of congestion by simple examination, and combined clinical and echocardiographic strategies may better define and reclassify the initial recognition. Notably, the early echocardiographic analysis may be additive in discerning the exact pathophysiology and in definition of AHF according to the most recent ESC guidelines, suggesting a simultaneous evaluation of perfusion or congestion status together with underlying triggering alteration (right heart failure, acute decompensated HF, pulmonary edema, and cardiogenic shock) (20).

### Previous reports

In 2019, a HFA multicenter survey dividing patients according to Stevenson criteria showed that the more represented groups were “wet and warm” (69.9%) and “wet and cold” (19.8%) whereas dry groups were less represented (21). Interestingly, in a subsequent analysis dividing patients according to six clinical profiles, patients with cardiogenic shock were “wet-cold” (54.8%), but a significant percentage was defined as “dry and cold” (26.4%) (22). Current findings reflect our subgroups percentage and adverse event rate; moreover, the different wet and cold group experienced the worse outcome whereas wet subgroups were prone to recurrent hospitalization. The echocardiographic analysis was performed in most of patients, but it did not specify the exact time frame and it was restricted to EF and mitral regurgitation assessment (22). In the same paper stratifying patients according to blood pressure quartiles, patients with systolic pressure below 85 mmHg revealed the worse prognosis.

### Gaps in clinical assessment

All these items suggest that physical examination not always identifies the real hypervolemic or euvolemic status (wet and dry), and the early recognition of congestion may be sometimes underestimated especially in those patients with occult fluid overload. Otherwise, the continuous right heart catheterization is capable of detecting right pressure and central venous pressure, but it does not always reflect the effective stroke volume and systemic fluid overload (12). Moreover, some doubts have yielded about the correspondence existing between invasive assessment of congestion/perfusion and the clinical profile according to the Stevenson picture (12, 23). The main limitation of this approach is related to the paucity of patients in warm and dry groups since in acute setting, it is unusual the recruitment of patients without congestion and hypoperfusion. Our sample reflects this trend and percentage of these patients is 2% of the whole population. Second, hemodynamic abnormalities partially explain the clinical features of AHF, but they do not completely account for the progression of congestion and perfusion when they are mediated by the prolonged activation of the renin angiotensin aldosterone system (RAAS) and of the sympathetic nervous system (SNS). Finally, current formulation does not discern between pulmonary and systemic congestion, which may occur independently (24). To avoid this bias, a recent ACVC position paper suggests to divide “wet” patients in pulmonary congestion resulting in acute respiratory failure, systemic congestion related to systemic volume overload, and tissue hypoperfusion leading to multi organ damage (25). Accordingly, our analysis highlights the concept that although Stevenson classification may be helpful to guide therapy across the initial phase and it provides important prognostic information, echocardiographic assessment during initial hospital admission phase may better recognize the effective clinical status adding important diagnostic features.

### Limitations

Our study was limited of small sample size and of restricted number of patients with a “dry-cold” profile precluding meaningful statistical analysis of this category, However, our classification is substantially in line with the wide ESC survey dividing patients according to Stevenson profile (21). This analysis retrospectively evaluated physical examinations performed as part of an observational study and classification were made by each investigator, this process may not have been readily reproducible or may have resulted in inconsistent classification, and this approach can potentially result in an inaccurate classification. Although we categorized patients based on the presence of two clinical signs, the reliability of classification needs further investigation. An adequate comparison between clinical and hemodynamic data to verify congestion or perfusion with invasive measurements is lacking (26). Echocardiographic examination was comprehensive of the most important parameters, although a detailed diastolic function analysis was not described. Therefore, systolic function analysis was limited to EF calculation, and stroke volume assessment was probably the more appropriate measurement to compare cold vs. warm patients. Despite the cited parameter is not included in the last position papers (15, 27), we believe that it could be added important insights on AHF and it is our intention to add this variable in the next analysis.

### Conclusion

The early Stevenson classification remains a simple and universally recognized approach for the detection of congestion and perfusion status, but the combined clinical and echocardiographic assessment should be extended in all patients presenting with AHF to better define the underlying pathophysiological alterations and possibly help in customized treatment. Extensive research that evaluates current and other available echo measurements should be warranted to confirm these preliminary findings.

### Take away message

Early echocardiographic assessment is an useful tool for better definition of patients with acute heart failure, and it adds new insights beyond Stevenson classification. Stevenson algorithm remains a hallmark for hemodynamic classification, although early echo assessment may better recognize specific pathophysiological features.

## Data availability statement

The raw data supporting the conclusions of this article will be made available by the authors, upon reasonable request.

## Ethics statement

Ethical approval was not provided for this study on human participants because it is a retrospective observational analysis. The patients/participants provided their written informed consent to participate in this study.

## Author contributions

All authors listed have made a substantial, direct, and intellectual contribution to the work, and approved it for publication.
